# Tick, tock, lock: night-time confinement in high security – history, practice, ethics and practicalities

**DOI:** 10.1192/bjb.2018.80

**Published:** 2019-02

**Authors:** Ed Silva, Andrew Shepherd

**Affiliations:** 1 Mersey Care NHS Foundation Trust, UK; 2 University of Manchester, UK; 3 Ashworth Hospital, UK.

**Keywords:** Forensic mental health services, in-patient treatment, psychiatry and law

## Abstract

**Summary:**

Night-time confinement, locking patients in their bedrooms overnight, is practiced within high-secure hospitals in the UK. This article provides context, sets out the history and reviews the ethical and pragmatic issues at stake. Thought is given to the future, where we appear to be moving toward a different approach.

**Declaration of interest:**

E.S. is a consultant forensic psychiatrist at Ashworth Hospital. All his patients are confined at night. He represents the Royal College of Psychiatrists Forensic Faculty at the National Oversight Group, which is the strategic advisory body providing assurance to NHS England regarding the commissioning and provision of high-secure services.

## History

Patients in UK hospitals are routinely locked up alone in their bedrooms from 21.15 h until 07.15 h for no clinical reason. This has recently been criticised by the Committee for the Prevention of Torture (CPT) in a report to the UK Government;^1^ hence this series of articles relating to practices in high-secure hospitals (Ashworth, Broadmoor, Carstairs and Rampton). Until the 1980s Blom–Cooper Inquiry,^2^ it was unremarkable that patients in the then special hospitals were locked in their rooms at night. The pendulum swung, and the 24 h opening introduced by one inquiry was questioned by the security review that followed another,^3^^,^^4^ which proposed night-time confinement (NTC) for ‘high-risk’ individuals – although this was rarely, if ever, used. As with almost all developments in forensic psychiatry, the reaction to a high-profile offence, followed by yet another inquiry introduced the Dangerous and Severe Personality Disorders services, which practiced NTC from their inception in 2003.

The current practice of NTC in the high-secure services has been permitted by the High Security Psychiatric Services (National Health Service Commissioning Board) Directions (current edition available through https://www.gov.uk/government/publications/high-security-psychiatric-services-directions). In 2011, the then Minister for Health, Andrew Lansley, first allowed the provider organisations to lock a patient's room at night only on the conditions that the room had a toilet and nurse call bell, or if the patient was continuously observed. No individual clinical reason to lock the door was required. Instead guidance required the decision to be made on the utilitarian argument that therapeutic benefit should be maximised for patients as a whole; for example, by releasing staff during the day. At the time, the chairs of the Royal College of Psychiatrists’ Forensic Faculty and Special Committee on Human Rights wrote to Mr Lansley to express the obvious concerns (*de facto* restriction of liberty through seclusion). The directions were considered by each of the provider trusts boards and NTC has been established in all the English high-secure hospitals as well as in Scotland (through different legal mechanisms), and has remained a subject for review within the existing governance arrangements. The recent Care Quality Commission (CQC) (https://www.cqc.org.uk/) reports remarked on the absence of available structured activity during the day for some patients in high-secure services, contrary to the original rationale. Significant further scrutiny has also followed the 2016 UK visit by the CPT who, among other things, said that ‘…the systematic locking-in of patients at night, which amounts to ten hours of *de facto* seclusion, is not acceptable in a care establishment provided there are sufficient staff.’^1^ (p. 75 para. 139).

In practice this means that, apart from the unrenovated wards in Rampton and Broadmoor, patients, with very few clinical exceptions, are locked in their rooms for 10 h per night, regardless of route to high security, offending history, current risk to others or dependency needs. It is quite possible for a patient to be subject to NTC in high security while awaiting transfer to low security or even, quite exceptionally, to a community placement. Although the guidance accompanying the directions requires clinicians to note the absence of contraindications to NTC, few if any psychiatrists in this invidious position do so (as evidenced by frequent discussions in peer supervision and other meetings); those that do not, find themselves in the curious position of being criticised by the CQC. Many, if not almost all, patients in high security have significant histories of self-harm or attempted suicide, and some present with ongoing extreme self-mutilation. At the time of writing, every patient in Ashworth is confined at night. Nursing staff now dispense night-time medication at 20.00 h and then muster patients to go to their rooms as the night shift arrives. Night-time staffing in the hospitals has significantly reduced, on occasion to levels that have been themselves seen as worryingly low. Curiously, most patients do not complain, either informally or otherwise, and some report feeling safer.^5^ At Forensic Faculty Committee meetings, it has been suggested that the obvious remedy is judicial review by a patient. This has not occurred.

## Research evidence

As for many coercive approaches in forensic practice,^6^ there is a significant absence of evidence in the literature. A search identified two mixed-methods studies.^7^^,^^8^ The first, based on the experience at Rampton, demonstrated no statistically significant change pre- or post-implementation in its chosen measures addressing ward atmosphere, working environment and patient quality of life. Semi-structured interviews suggested staff observed a less negative effect than had been anticipated, whereas patients expressed a certain ambivalence. The findings from the study are potentially influenced by the close affiliation between researchers and the site of study, as well as by the choice of wards (acute admission) for the pilot. In the second study, based on the experience at Ashworth, measures were collected relating to sleep hygiene, behaviour and findings from a bespoke questionnaire, seeking to understand patients' concerns regarding NTC. No statistically significant changes were observed. However, a parallel investigation considering the attitudes of nursing staff revealed a universally negative attitude to it. The potential to generalise findings from the study were limited by difficulties in recruitment and implementation, which may have resulted in skewed findings in relation to the experience within the hospital as a whole. More generally, as noted above, there is a general absence of evidence in relation to the implementation of practices such as seclusion. A qualitative study, addressing the process of seclusion in forensic practice,^9^ identified clear communication of the ‘purpose’ of seclusion as being a key ingredient in managing distress associated with the experience. Widespread application of NTC could be seen as partially mitigating this factor if it is assumed to be a normal practice by patients, staff and others.

## NTC and values-based practice

Building on the concept of principled ethical clinical practice, the Francis report^10^ has reiterated the call for care providers to develop a culture that focuses first on the patient and providing, within resource limitations, compassionate care that does not inadvertently restrict basic rights or cause avoidable harm.

The CPT report notes that NTC had the potential to cause individual anxiety and that there was a lack of evidence of individual risk assessment to mitigate this potential distress. Although the limited research evidence base surveyed suggests an absence of harm, the CPT also noted the absence of evidence of benefit and the CQC reports are mixed, again highlighting concern at the restriction of liberty and remarking on a lack of increased daytime structured activity by way of mitigation.

Individual autonomy is clearly restricted through the act of confinement at any time. However, arguments in relation to autonomy are complicated with mentally disordered offenders^11^ because acts of violence against individuals or society more generally lead to a socially sanctioned act of imprisonment. Although the situation of forensic in-patients will vary, the majority are not subject to court-ordered punishment. This may add weight to the position outlined in the Tilt report,^4^ which proposes that specific ‘high-risk’ individuals may perhaps be ‘proportionately’ subject to NTC. The blanket application of this restriction regardless of risk, or progress along the care pathway, is confusing to many.

The argument raised in support of NTC is that of a justice-based position, whereby resources consumed in the staffing of night shifts, to allow free movement of patients, can be more appropriately allocated in the day, to increase the availability of therapeutically oriented activity. However, as has been noted by both the CPT and the CQC, there has been an absence of increased structured activity during the day even with NTC, suggesting that no benefit has emerged in this area. However, interpretation of this situation is complex because, with the notable increase in constraint on resources in recent years, it is hard to determine how services would currently appear had funding remained on the projected trajectory from the time of NTC's inception.

## Moving forward

The current position with regard to the practice of NTC is therefore difficult, particularly in its current blanket implementation. There is also a pragmatic reality: even if the decisions were reversed, neither the money nor the staff are available to fill the gaps.

To return to the analogy of the pendulum, swings between restrictive and more liberal practice can be seen as an institutional group response to anxiety and external scrutiny.^12^^,^^13^ Generally, these changes are seen as being a collective response from within the group; however, in this situation the swing of the pendulum has been affected by the massive gravitational change of austerity. Clinical decision-making is forced, in that it is being subjected to either political pressure or fiscal reality, depending on one's viewpoint. As in other political arenas, it seems apparent that it is some of the most vulnerable in society who are subjected to restriction. There is also an ‘invisibility’ to the phenomenon, occurring as it does behind the opacities of our walls, and it is perhaps also curious that it took an investigation from the CPT, rather than our own governance structures, to switch the night light on, and so we should thank our European friends. Movement to a more dynamic and rational response is necessary. The pendulum must swing again, but how can this best be achieved?

## About the authors

**Ed Silva** is a consultant forensic psychiatrist with Mersey Care NHS Foundation Trust, UK; **Andrew Shepherd** is a clinical lecturer in forensic psychiatry at the University of Manchester, UK and is a SpR in Forensic Psychiatry at Ashworth Hospital, Mersey Care NHS Trust, UK.
